# Supplementary material for: Effects of age on the identification of emotions in facial expressions: a meta-analysis
Source: PeerJ. 2018 Jul 25;6:e5278. doi: 10.7717/peerj.5278 (PMC6064197; doi:10.7717/peerj.5278)
Supplement: Supplemental Information 2 [file peerj-06-5278-s002.docx]

The requested information is presented below.

1. The rationale for conducting the meta-analysis:

Although it is well established that a general cognitive decline occurs with advancing age and several studies addressed the effects of aging on emotion recognition abilities, questions about the impact of aging on emotional processing persist. A meta-analysis by Ruffman and colleagues (2008) explored this issue, but much research has been published since then, reporting inconsistent findings.

1. The contribution that the meta-analysis makes to knowledge in light of previously published related reports, including other meta-analyses and systematic reviews:

The present meta-analysis shows less accuracy of older adults in emotion identification not supporting a positivity bias nor a reduction in the negativity effect, and strengthening the results obtained in a previous meta-analysis by Ruffman et al. (2008). The meta-regression analysis indicate that effect sizes are moderated by sample characteristics such as sex and level of education.
